# Plant Foods Rich in Antioxidants and Human Cognition: A Systematic Review

**DOI:** 10.3390/antiox10050714

**Published:** 2021-04-30

**Authors:** Luciana Baroni, Anna Rita Sarni, Cristina Zuliani

**Affiliations:** 1 Scientific Society for Vegetarian Nutrition, I-30171 Venice, Italy; anna.sarni@scienzavegetariana.it; 2 Neurology Unit, Mirano Hospital, I-30035 Mirano, Italy; vdf@protec.it

**Keywords:** antioxidants, phytochemicals, polyphenols, flavonoids, oxidative stress, cognition, cognitive impairment, neurodegeneration, aging

## Abstract

Oxidative stress can compromise central nervous system integrity, thereby affecting cognitive ability. Consumption of plant foods rich in antioxidants could thereby protect cognition. We systematically reviewed the literature exploring the effects of antioxidant-rich plant foods on cognition. Thirty-one studies were included: 21 intervention, 4 cross-sectional (one with a cohort in prospective observation as well), and 6 prospective studies. Subjects belonged to various age classes (young, adult, and elderly). Some subjects examined were healthy, some had mild cognitive impairment (MCI), and some others were demented. Despite the different plant foods and the cognitive assessments used, the results can be summarized as follows: 7 studies reported a significant improvement in all cognitive domains examined; 19 found significant improvements only in some cognitive areas, or only for some food subsets; and 5 showed no significant improvement or no effectiveness. The impact of dietary plant antioxidants on cognition appears promising: most of the examined studies showed associations with significant beneficial effects on cognitive functions—in some cases global or only in some specific domains. There was typically an acute, preventive, or therapeutic effect in young, adult, and elderly people, whether they were healthy, demented, or affected by MCI. Their effects, however, are not attributable only to anti-oxidation.

## 1. Introduction

Although there is no consensus, healthy aging is not just the absence of cognitive impairment, but also the preservation of all human abilities, including social connectedness, proper responses to environmental challenges, an ongoing sense of purpose, and so on [1]. In the last century, the increase in size of the aging population translated into globally higher rates of age-related cognitive decline (ARCD) and dementia. Non-modifiable and modifiable risk factors associated with dementia include advanced age, low level of education, female gender, genetic susceptibility, obesity, insulin resistance, hypertension, smoking and other cardiovascular risk factors, head trauma, and drugs, whereas cognitive enrichment early in life promotes cognitive reserve [2,3,4,5,6,7].

Oxidative stress (OxS) may derive from mitochondrial dysfunction, inflammatory status, heavy metals, amyloid-(A), and hyperphosphorylated tau accumulation [8]. Chronic systemic OxS stimulates a cascade of reactive oxygen species (ROS) and reactive nitrogen species (RNS), which impairs the blood–brain barrier (BBB), and contributes to lipid peroxidation, oxidation of proteins and nucleic acids, brain cell damage, and even cell death. Nevertheless, a thin line separates the harmful from beneficial ROS effects, depending on redox balance: they not only act as damaging agents, but also participate in cellular signaling and regulation [9]. In fact, polyphenol benefits have been partly attributed to their pro-oxidant activities [10,11]. Evidence shows oxidative imbalances in MCI and Alzheimer’s disease (AD) and other neurodegenerative disorders, with lower peripheral levels and activities of antioxidants as compared to controls [4,12]. OxS markers are commonly increased in neurodegenerative disorders. AD patients show higher levels of urinary oxidized products of proteins and DNA and abnormal OxS markers [12,13,14,15,16]. MCI is associated with an increased risk of dementia, particularly AD, and brains of MCI patients share many pathological features with those of AD subjects [17]. MCI and AD patients have shown similarly lower levels of plasma antioxidants, while higher levels were documented in healthy, human centenarian blood [12,18,19].

For our systematic review, we selected only studies in which the food was used as an established source of antioxidant molecules. Nevertheless, OxS and inflammation play interconnected and mutually reinforcing roles in cognitive decline and neurodegeneration [20,21]. As a matter of fact, some antioxidant compounds can influence cognition through further mechanisms, other than their protection against OxS.

Dietary patterns centered on antioxidant-rich plant foods (the Mediterranean diet, the Dietary Approaches to Stop Hypertension Trial (DASH) diet, and the Mediterranean-DASH Intervention for Neurodegenerative Delay (MIND)) are effective dietary strategies to maintain cognitive health; improve overall global cognitive function; and prevent ARCD, MCI, and the progression from MCI to AD [3,4,22,23,24,25,26,27]. The only study performed on vegetarians found that in a group of over 3000 individuals belonging to two distinct substudies (272 matched and 2984 unmatched subjects), the matched non-vegetarian cohort had twice the risk of developing dementia compared to vegetarians, whereas there was no significant difference in the incidence of dementia in the vegetarian versus meat-eating unmatched subjects. However, there was a trend towards delayed onset of dementia in vegetarians in both substudies [28].

Among the plant foods shown to positively influence cognitive functioning, there are cruciferous vegetables, green leafy vegetables, carrots, oranges, apples, nuts, spices, berries, grapes, pomegranates, chocolate, coffee, blue potatoes, onions, olive oil, and seaweed [4,19,29,30]. Most, but not all, of these foods have been described in relation to cognition. Moreover, among all the antioxidant compounds, the only dietary antioxidant class that has been investigated is polyphenols.

Interestingly, administrating “isolated” plant antioxidants as supplements has failed to prevent chronic diseases. This could be related to the different effects of most antioxidants when they are taken out of their “natural food environments.” As a matter of fact, plant food contains thousands of different phytochemicals with various activities, capable of synergistic actions either as direct antioxidants or via gene expression modulation. Hence, it is plausible that their orchestrated activities are not replaceable by a single, or a combination of extracted phytochemicals [19,31].

Table 1 summarizes the characteristics of the major phytochemicals contained in the plant foods described in the results of this systematic review, in order to provide a quick summary of their presence and activity. As can be observed, the plant foods selected for our review as rich sources of antioxidants contain an array of different molecules with antioxidant properties and other properties, whose isolated and synergistic effects cannot be easily detected nor distinguished from each other. This situation is responsible for the bias that affects this kind of study, and that should be always kept in mind when evaluating the results.

This paper aims to review the state-of-art of this topic, thereby describing the emerging findings relating the intake of some plant foods rich in antioxidant polyphenolic molecules with cognitive status. As will be highlighted, most of the studies found significant associations between the intake of the examined foods and beneficial effects on cognitive functions.

## 2. Materials and Methods

We performed PubMed/MEDLINE (https://pubmed.ncbi.nlm.nih.gov/ Accessed date 16 October 2020), ScienceDirect (https://www.sciencedirect.com/ Accessed date 15 October 2020), and Cochrane Trials Library (https://www.cochranelibrary.com/central Accessed date 15 October 2020) systematic database searches, ending 18 October 2020, using the following items in combination:

(a) “plant-based” OR “vegan” OR “veganism” OR “vegetarian” OR “vegetarianism” OR “adventist”;
(b) “neurodegeneration” OR “dementia” OR “Parkinson” OR “cognitive disease” OR “extrapyramidal” OR “extrapyramidalism” OR “neurodegenerative” OR “cognition” OR “Alzheimer”;
(c) “oxidative” OR “antioxidant” OR “phytochemical” OR “ROS.”

The following filters were applied for all items: English language, title, abstract. Additionally, for (c) AND (a) AND (b), filters were applied for: clinical trial, randomized controlled trial, and clinical studies.

The research yielded 2205 records: 428 from Pubmed, 1667 from Cochrane Library, and 71 from Sciencedirect; 39 other references were extracted according to query criteria from available reviews or other sources. After the removal of duplicates, 2111 records were evaluated for inclusion on the basis of the following criteria:

Inclusion criteria

- Observational or intervention study design.
- Sufficient definition and data of the food source or dietary antioxidant intake.
- Data on cognition assessments of normal subjects and subjects affected by the main neurodegenerative diseases compromising cognition.

Exclusion criteria

- Case reports, reviews, metanalyses.
- In vitro or animal studies.
- Co-administration of drugs affecting CNS.
- Supplements not consumed in the form of food.
- Insufficient data on cognition measurements.

The outcomes of each researcher were reported independently, and differences were discussed until agreement was reached. The selection led to the exclusion of 2003 records, including 2 that were not in English. One hundred eight full-texts of the selection were retrieved, and further filtering was performed by reading each full-text to verify its inclusion in the final selection. After full-text screening, 77 articles were excluded: 2 were not found in full-text form, 31 were reviews or meta-analysis, 5 were in-vitro or animal studies, 1 reported co-administration of drugs affecting CNS, 31 administered supplements not consumed in the form of food, and 7 reported insufficient or no data on cognitive outcomes.

Of the final 31 articles, 4 were cross-sectional studies (1 of them also had a cohort in prospective observation), 6 were prospective studies, and were 21 intervention studies (20 RCTs and 1 open-label pilot study).

**Figure 1:**
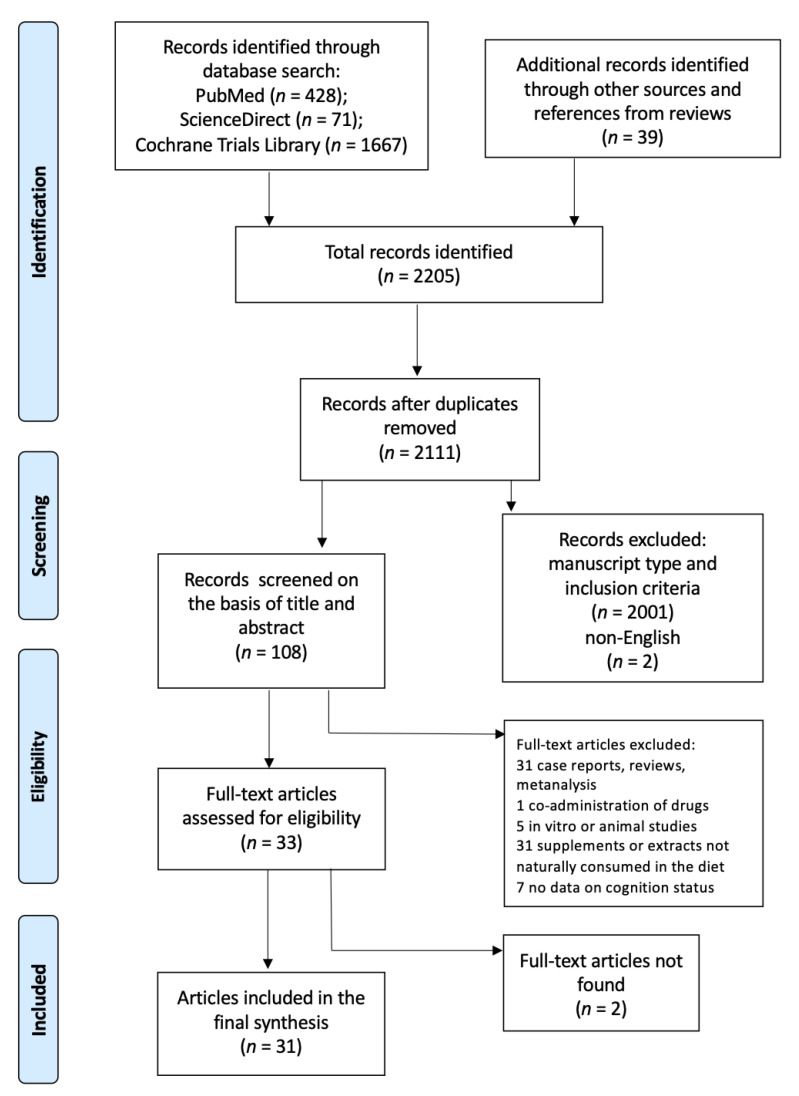
Flow diagram of the search strategy. Adapted from Moher et al., 2009 [68].

The procedures were carried out following the guides released by The PRISMA Group [68]. The flow chart in Figure 1 shows the steps of the process.

## 3. Results

Thirty-one studies published between 2006 and 2020 met the inclusion criteria for the review, with a large clinical and methodological heterogeneity, resulting from variability of the characteristics of the study sample, the plant-based source of antioxidant, the study design and duration, and the choice of cognitive outcomes used. The findings are therefore presented by grouping the studies that used the same plant-food sources.

Regarding the great heterogeneity of cognitive assessments, which were, however, performed in all studies using standardized, referenced, and validated tests: in our review we decided not to specify the name of every test performed in each study, but only the cognitive domains analyzed.

Finally, since our review focuses on the cognitive outcomes of the intake of dietary natural plant foods, we did not investigate data on neuroimaging correlates or on oxidative stress markers, which were present in some of the studies considered.

### 3.1. Nuts

On this topic we found six studies investigating the impact of nuts on cognition: 1 prospective cohort study [36], 1 cross-sectional study (including two groups of subjects of different ages) [37], and 4 RCTs [32,33,35,40], among which one study was performed in association with olive oil and the Mediterranean Diet [33].

In the 6-year substudy of the prospective cohort Nurses’ Health Study (NHS), including 15467 women who were 70 years-old [36], higher, long-term total nut intake was associated with better average cognitive status for all the cognitive outcomes, encompassing global cognition and verbal memory. In the global, composite score, combining all tests, women consuming at least five servings of nuts/wk had higher scores than non-consumers (*p* = 0.003), but long-term intake of nuts was not associated with rates of cognitive decline.

In a cross-sectional study of two groups of adults (20–59 years-old, *n* = 5356; and 60 years-old and older, *n* = 7337) participating in the National Health and Nutrition Examination Surveys (NHANES), walnut consumption [37], visuo-motor speed, information-processing speed, concentration, motor control, learning, and recall were assessed; in the older group, attention and delayed verbal memory were also assessed. Subjects in the younger group, reporting a walnut consumption of an average of 10.3 g/d, required significantly less time to respond to the visuomotor speed test (*p* = 0.03) and to the information processing speed test (*p* = 0.01), and had significantly lower time scores for learning and recalling (*p* = 0.05). Significantly better outcomes were also recorded in all cognitive test scores among those with higher walnut consumption (*p* < 0.01). Subjects in the older group, consuming an average 13.1 g/d of walnuts, scored significantly higher in the attention-assessing test (*p* = 0.03) and verbal memory (*p* = 0.05).

A parallel group RCT randomly assigned 334 subjects, 55–80 years-old and at high cardiovascular risk, to one of three groups: a Mediterranean diet supplemented with extra virgin olive oil (MO); a Mediterranean diet supplemented with 30 g/d of mixed nuts (MN), and a control diet (CD) [33]. After a median of 4.1 years, MN participants scored better on tests evaluating immediate and delayed episodic memory (*p* = 0.04), and in the memory subtests of global cognitive composites compared with CD participants (*p* = 0.04); no between-group differences were observed for the other cognitive tests.

Another single blind, parallel-group RCT, performed in two centers (Loma Linda and Barcelona), evaluated the effects of a diet enriched with walnuts to 15% of daily energy intake compared with a diet without walnuts in 657 cognitively healthy subjects 63–79 years-old [32]. No participant developed a clinically significant impairment in the global cognitive composite score or memory, language, perception, and frontal functions during the 2-year follow-up. Nevertheless, post-hoc analyses showed that only walnut-diet arm participants, from the Barcelona site, statistically improved in global cognition and perception scores compared with controls (*p* = 0.04).

A total of 64 college students were randomly assigned to two treatment sequences in a double-blind, crossover, placebo RCT: walnuts–placebo or placebo–walnuts [40]. The test meal (banana bread with 60 g/d of walnuts) and the placebo meal (banana bread without walnuts) were consumed for 8 wks, separated by 6 wks of washout. Non-verbal reasoning, verbal reasoning, and memory were assessed at baseline and at the end of each of the two 8-wk treatment periods. No significant increases were detected for non-verbal reasoning or memory with the walnut-supplemented diet. However, inferential verbal reasoning increased significantly by 11.2%, indicating a medium effect size (*p* = 0.009).

A placebo RCT that enrolled 31 subjects with MCI randomly assigned participants to an intervention group—to consume one Brazil nut each day for 6 mo (*n* = 16); or to the control group—avoiding Brazil nuts during the study (*n* = 15) [35]. Changes in the total scores of fundamental cognitive abilities were not significantly different between groups after the intervention period in comparison with baseline. However, improvements in verbal fluency (*p* = 0.007) and constructional praxis (*p* = 0.031) were significantly greater in the intervention group when compared with the control group.

### 3.2. Fruit and Vegetables

We found nine RCTs and one open-label pilot study in which the intervention antioxidant-rich food was a single fruit in the form of juice [30,47,48,49,52,53,54,56,69,70]; in three additional RCTs a reconstituted drink was used [41,46,71]; two more RCTs described the intake of a single vegetable [59,72]; and two other prospective studies used fruit and vegetable (and nuts) intake together [73] or a single beverage [74]. Finally, two more studies were focused on analyzing flavonoid intake prospectively [75] and after intervention [76]. In the following descriptions, the studies are grouped on the basis of the foods examined.

#### 3.2.1. Grapes (From Different Cultivars)

A double-blind, counterbalanced-crossover placebo RCT assessed the cognitive benefits of purple grapes in a sample of 20 healthy young adults [47]. The active treatment consisted of 200 mL Welch’s Purple Grape Juice (IG), while the placebo consisted of 200 mL Welch’s White Grape Juice (CG). Each participant underwent two sessions 7-d apart, to allow for washout. Cognitive outcomes of memory tasks (immediate and delayed word recall, numeric working memory, word recognition, and picture recognition) and attention tasks (simple reaction time, choice reaction time, and digit vigilance) were calculated. These were then further subdivided into four composite scores on the basis of whether they measured accuracy (percentage accuracy) or reaction time (ms). IG significantly improved reaction time in the composite attention measure (*p* = 0.047), when compared to CG. Order effects also indicated an enduring positive effect on pre-dose memory reaction time (*p* = 0.018) when purple grapes were consumed first.

A double-blind, placebo RCT was performed for at least 6 months on 10 subjects aged 66–82 years-old (mean age 72.2 years-old), with MCI [41], randomized to consume, twice daily, either an active grape formulation or a placebo formulation. Patients underwent a complete neuropsychological battery evaluating all cognitive domains at baseline and at 6 months. No significant benefits for the active formulation arm were noted in scores from the assessments of the neuropsychological battery.

Another double-blind, placebo RCT enrolled 12 subjects (mean age 72.8 years-old) with early-memory changes (not dementia) [30]. Five subjects were randomly assigned to assume Welch’s Foods 100% Concord Grape Juice, and seven subjects received the placebo every day for 12 wks. Covariance analysis demonstrated a significant positive effect (*p* = 0.04) in learning trials, and positive, but not statistically significant, trends toward improvement, with respect to delayed verbal recall and spatial memory in the Concord Grape Juice group, compared to placebo.

#### 3.2.2. Berries

In a successive double-blind, placebo RCT, with the same design as the previous study [30], nine elderly MCI participants (mean age 76.2 years-old) assumed between 6 and 9 mL/kg/d wild blueberry juice for 12 wks [48]. The placebo beverage was the same as that of the previous study [30]. Significant improvements in the paired associate learning task (*p* = 0.009) and word list recall (*p* = 0.04) were observed after intervention. However, the strength of both of these studies is limited by the small sample sizes [30,48].

In a double blind, placebo RCT [49], 26 healthy elderly subjects were randomized: 12 to consume 30 mL/d of blueberry concentrate for 12 wks, and 14 to consume an isoenergetic placebo. The results highlighted a non-significant improvement, only for working memory, in the intervention group.

Two groups of 20 healthy volunteers aged 50–70 years-old were included in a crossover RCT [46]. They consumed the intervention beverage consisting of Swedish berries for 5-wks and then the control beverage, in inverse order, separated by a 5-wk washout. The effects of the intervention on the working memory capacity, selective attention, and psychomotor reaction time were evaluated. Subjects performed better only in the working memory test after intervention (*p* < 0.05); no significant effects on the other test variables were observed.

A last double-blind, placebo RCT investigated the effects of dietary blueberries on the mobility and cognition of 37 subjects aged 60–75 years-old [71] against a placebo. Eighteen subjects consumed 12 g of a standardized blend of Tifblue blueberries twice a day for 90 d. Executive function, long and short-term memory, spatial cognition, and attention were evaluated at baseline and after 45 and 90 d of intervention. Subjects in the intervention group showed a significant reduction in reaction times (*p* < 0.001), fewer repetition errors in the long term memory test (*p* = 0.031), and reduced switch cost on a task-switching test (*p* = 0.033) in comparison to the controls.

#### 3.2.3. Cherries

A 12-wk, placebo RCT, conducted on 42 elderly subjects with mild-moderate dementia, administered cherry juice to the intervention group (*n* = 21), and commercially prepared apple juice to the control group [69]. Verbal learning and memory, working memory, semantic memory, short-term memory, executive function, and executive control processes were assessed at baseline, and at 6 and 12 wks. At the end of the study, the intervention group showed significant improvements in verbal fluency (*p* = 0.014), short-term memory (*p* = 0.014), and long-term memory (*p* ≤ 0.001) when compared to the control group.

Another 12-wk, placebo RCT involving 34 adults aged 65–80 years-old with normal cognitive function, randomly assigned two groups to consume 2 cups/d of a commercially available Montmorency tart cherry concentrate (*n* = 17) or placebo (*n* = 17), for 12 wks [52]. After the 12-wk period, the intervention group had higher memory scores (*p* = 0.02) and performed better on the learning task (*p* = 0.02) compared to the control group. The within-group analysis showed a significant improvement in visual sustained attention (*p* < 0.0001) and spatial working memory (*p* = 0.06) after intervention, compared with the corresponding baseline values.

#### 3.2.4. Pomegranate

A double-blind, two-group, parallel RCT evaluated memory performance in 200 subjects, aged 50–75 years-old, with normal aging or MCI [53]. Ninety-eight subjects were randomized to the intervention group—8 oz/d (236.5 mL) of pomegranate juice, and the 102 subjects in the control group consumed a placebo drink. Visual memory and visual learning and recall were assessed at baseline and after 6 and 12 mo. Visual memory performance, specifically the ability to learn visual information over repeated learning trials, was shown to be maintained during the observation time in individuals who drank pomegranate, whereas the placebo group showed a significant decline (*p* = 0.002).

These findings are consistent with a preliminary placebo RCT published 6 years earlier by the same group [54], which randomized 28 non-demented, elderly subjects to consume 8 oz/d of pomegranate juice or a placebo for 4 wks. Each subject underwent brief visual and verbal memory testing immediately prior to beginning the trial (t1) and at 28-d of pomegranate juice or placebo (t2). The within-group analysis of changes found that the intervention group performed significantly better at t2 compared with t1 (*p* = 0.017), whereas there was no difference for the control group. Between groups, the t2 versus t1 improvement in memory scores was significantly greater, in the total recall measure, in the intervention group compared to the control group (*p* = 0.029). Similarly, in the consistent long-term retrieval test, the intervention group recalled more items compared to the controls (*p* = 0.022).

#### 3.2.5. Oranges

Thirty-seven healthy elderly subjects (range 60–81 years-old, mean 66.7 years-old) were enrolled in a double-blind, crossover, placebo RCT [56]. Participants consumed a naturally high flavanone (HF, 305 mg) 100% orange juice or an isocaloric, low flavanone (LF, 37 mg) orange-flavored cordial (500 mL) each day for two 4-wk periods, separated by a 4-wk washout, in inverse order. Participants were tested every 4 wks. No significant difference was observed between the two groups in the global cognitive performance at baseline, whereas the performances and the executive functions were significantly better after intervention (*p* < 0.05 for both). The drink sequence did not significantly affect the vast majority of outcomes, but better performances were observed in sustained attention and episodic memory when the HF drink was consumed first.

#### 3.2.6. Apples

An open-label pilot study recruited 21 nursing home residents who were 72–93 years-old (mean 82 years-old) and affected with moderate-to-late stage AD [70]. Each participant consumed 24-oz/d of apple juice for 1 month, and completed at baseline and after 1-mo validated testing, assessing cognitive performance. Behavioral and psychological symptoms of dementia (BPSD) were also evaluated. No change in cognitive performance was detected over 1-mo interval; by contrast, caregivers reported significant (*p* < 0.01) improvement in BPSD.

#### 3.2.7. Onion

A double-blind, placebo RCT examined the effects of quercetin-rich onions on the inhibition of cognitive decline in 50 volunteers aged 65–84 years-old, healthy or with MCI [59]. Subjects consumed 10 g/d of either an active onion powder containing 60 mg of quercetin or a placebo powder. The Mini-Mental State Examination (MMSE) [77], which includes questions on orientation in time and place, registration, attention and calculation, recall, language, and visual construction;, and other cognitive testing assessing executive function, memory recall, immediate memory, and short-term memory, were performed at baseline, and 12 and 24 weeks after the beginning of the study. Cognitive assessment showed no significant differences between the intervention and the placebo groups from the baseline to the other time points. However, in younger subjects, the MMSE scores were significantly higher in the intervention group after 24 wks (*p* = 0.019).

#### 3.2.8. Rosemary

A double-blind, repeated-measure, crossover, placebo RTC investigated the acute effects of 100% powdered rosemary (*R. officinalis* L.) in 28 healthy and non-smoking elderly subjects aged 65–90 years-old (mean 75 years-old) [72]. Attention, working memory, episodic memory, and “speed of memory” were evaluated at 1, 2.5, 4, and 6 h, following four different doses of rosemary, and a placebo. The tests were performed in five separate treatment sessions, every week for five weeks. There was a dose-dependent effect on measures of “speed of memory”: the lowest dose (750 mg) of rosemary had a statistically significant, beneficial effect, compared with placebo (*p* = 0.01), whereas the highest dose (6000 mg) had a significant detrimental effect (*p* < 0.01). There were significant but less consistent deleterious effects on other measures of cognitive performance compared with baseline, for the placebo and all treatments except the 750 mg dose.

#### 3.2.9. Mixed Plant Sources

In a prospective study, the cognitive function of a general population sample of 2613 subjects, aged 43–70 years-old at time of enrollment, was examined at baseline, and after five years [73]. At baseline, a validated, self-administered, semi-quantitative questionnaire was used to assess the habitual consumption of 178 food items during the previous year. The total intakes of four main food groups (fruits, vegetables, legumes, and juices) were calculated. The neuropsychological assessment included a battery of validated tests measuring global cognitive function (all cognitive tests combined) and specific cognitive domains, such as memory function, information processing speed, and cognitive flexibility. Higher reported vegetable intake was associated with lower information processing speed (*p* = 0.02) and worse cognitive flexibility (*p* = 0.03) at baseline, but with a smaller decline in information processing speed (*p* < 0.01) and global cognitive function (*p* = 0.02) at follow-up. High intakes of some subgroups of fruits and vegetables (such as nuts, cabbage, and root vegetables) were associated with better cognitive function at baseline and/or smaller declines in cognitive domains. In particular, higher intake of nuts was associated with better cognitive function at baseline in all domains (all *p* < 0.01), and the difference in cognitive function between the lowest and the highest quintiles was equivalent to a 5–8-year difference in age. Moreover, higher intake of cabbage was associated with better memory function (*p* < 0.02) and better global cognitive function (*p* < 0.01) at baseline, and with a smaller decline in information processing speed at follow-up (*p* < 0.01). At follow-up, individuals in the lowest quintiles of cabbage consumption showed about two times the decline in information processing speed of the individuals in the highest quintile.

Using a cohort of 1589 dementia-free subjects 65 years-old and older, included in the Kame Project, a large, population-based, prospective study of Japanese Americans in King County, Washington, Dai et al. followed participants from 1992 to 2001 [74]. The participants at baseline filled out a self-administered, semi-quantitative, food frequency questionnaire. Fruit and vegetable juice intake frequency was classified as “<1/wk,” “1–2/wk,” or “≥3/wk.” Moreover, participants were assessed with a cognitive screening test at baseline and at each of four follow-up timepoints, each two years apart for a total of four incidence timepoints. After adjustment for potential confounders, the risk for probable AD was significantly reduced among people who drank fruit and vegetable juices 1–2/wk, and even more so for ≥3/wk, compared with those who drank these juices <1/wk (*p* < 0.01).

#### 3.2.10. Flavonoids in Food

In a prospective study, the dietary habits of 808 southern Italian adults aged 50 years-old or over were analyzed [75]. Data on the flavonoid content in foods were retrieved from food frequency questionnaires, and the total mean intakes of, the subclasses of, and the individual flavonoids involved were calculated. A significant inverse association between higher dietary intake of total flavonoids and impaired cognitive status was found. Among individual subclasses of flavonoids, flavan-3-ols, catechins, anthocyanins and flavonols, and only quercetin among individual polyphenols, were associated with cognitive health. A significant difference between individuals with normal and impaired cognitive function was found only in the dietary intake of anthocyanins (*p* = 0.017).

A crossover RCT performed on 30 healthy volunteers (mean age 47.3 years) investigated the acute effects of apples and spinach (rich in nitrate) independently, and in combination with nitric oxide (NO) status, cognitive function, and other indicators [76]. Cognitive function was tested using a tailored version of a cognitive assessment battery measuring “quality working memory”, “power of attention”, and “continuity of attention”. Each participant completed four visits that evaluated cognitive function measures for 150 min post-lunch, with a minimum 1-wk washout period between visits. No significant effect was observed on cognitive function, and no relation was found with NO status.

### 3.3. Caffeinate Foods

We found six studies investigating the impacts of caffeine-containing food intake on cognition: one cross-sectional study evaluated the effectiveness of chocolate alone [78], one prospectively treated the effectiveness of coffee and tea together [63], and another considered prospectively the effectiveness of coffee and chocolate together [79]; three studies focused instead on the effectiveness of tea consumption: one placebo RCT [80], one cross-sectional plus prospective study [81], and one cross-sectional study [82].

#### 3.3.1. Chocolate

Chocolate intake was evaluated in 968 non-demented, community-dwelling subjects aged 23–98 years-old, participating in a cross-sectional study by Crichton, a cohort of the Maine-Syracuse Longitudinal Study (MSLS), a community-based study of cardiovascular risk factors and cognitive functioning in adults [78]. Dietary intake was assessed using a validated questionnaire, without differentiating according to the type of chocolate. Global composite score, visual-spatial memory and organization, scanning and tracking, verbal episodic memory, and working memory were assessed. More frequent chocolate consumption was significantly associated with better performance in all tests (*p* < 0.05), with the exception of working memory, and these relations were not attenuated after statistically controlling for cardiovascular, lifestyle, and dietary factors.

The 145 elderly subjects included in the study by Haller represented a cohort retrieved from a large, population-based, prospective study on healthy aging that was still ongoing in the Geneva and Lausanne counties at the time of publication [79]. Usual caffeinated food and beverage (coffee and chocolate) consumption, and wine intake, were assessed by a self-administered questionnaire. At baseline, all individuals underwent complete assessments of the following cognitive domains: attention, working memory (verbal and visuo-spatial), episodic memory (verbal and visual), executive functions, language, visual gnosis, and praxis. Two neuropsychological assessments during a 3-year follow-up (18 and 36 months after inclusion) allowed the classification of subjects according to the progression trend. After adjusting for age, sex, education level, and cognitive testing, only wine was associated with an increased risk of adverse evolution (*p* = 0.028). When analyzing the consumption data in tertiles, moderate coffee drinkers were less likely to be classified in the worse stage (*p* = 0.037). This observation persisted after adjusting for wine and chocolate consumption (*p* = 0.048).

#### 3.3.2. Tea and Coffee

A community-based observational study (cross-sectional study plus prospective study) analyzed a cohort of 2194 community-living Chinese adults aged >55 years-old without cognitive impairment at baseline [81]. Detailed information on tea and coffee frequency consumption was collected at baseline, and cognitive function was reassessed after 1–2 years (median: 16 months) in 1438 subjects (65.5%) using the MMSE. Total tea intake was significantly associated with a lower prevalence of cognitive impairment (*p* < 0.001) and was most evident for black (fermented) and oolong (semi-fermented) teas, the predominant types consumed by this population. In contrast, no association was found between coffee intake and cognitive status.

Consumption of coffee and tea were assessed in a population-based prospective study that included 1409 subjects who underwent re-examination after an average follow-up of 21 years [63]. They had a mean age of 50.4 years-old at the midlife examination, and 71.3 years-old at the late-life examination. Cognitive status was assessed using a three-step protocol for the diagnosis of dementia (a screening, a clinical, and a differential diagnostic phase). The consumption of coffee and tea had been assessed quantitatively at the midlife examination. Coffee drinking was categorized into three groups. Furthermore, tea consumption was dichotomized into non-tea drinkers (0 cup/d) and tea drinkers (>1 cup/d). In later life, the low coffee consumers (0–2 cups/day) had the highest occurrence of dementia and AD, and the lowest risk (65–70% decreased) was found in people who drank 3–5 cups/d. Tea consumption had no associations with dementia or AD.

A cross-sectional study from a community-based comprehensive geriatric assessment (CGA) analyzed 1003 Japanese subjects aged >70 years-old [82]. Data about the frequency of recent consumption of five beverages (green tea, black or oolong tea, coffee, cola, juice, or 100% fresh vegetable juice) and 55 items related to food intake during the previous month were collected. Cognitive function was tested using the Japanese language version of the 30-point MMSE. Statistically significant inverse associations were observed between green tea consumption and cognitive impairment (*p* = 0.0006), with a gradient with different frequencies of consumption. In contrast, a weak or null association was observed between intake of black or oolong tea or coffee and the prevalence of cognitive impairment.

Finally, a placebo RCT evaluated the effect on cognition of green tea consumption in 33 elderly nursing home residents with cognitive dysfunction [80]. The participants were randomized to consume 2.0 g/d of green tea (*n* = 17) or placebo (*n* = 16) for 12 months. Tests for assessing cognitive function were administered at baseline and every 3 months. Changes in cognitive scores of the intervention group after 12 months were not significant.

Table 2 displays the studies’ characteristics and the main findings of the articles included in the systematic review.

## 4. Discussion

Our systematic review investigated the effects of antioxidant-rich plant foods on human cognitive functions. Since we did not find any studies that clearly showed measurable activities of natural plant antioxidants, other than polyphenols, this review’s focus was on naturally occurring dietary polyphenols. Table 3 proposes a simple classification of naturally occurring plant polyphenols.

Polyphenols represent the largest group of phytochemicals that directly scavenge superoxide and hydroxyl radicals, peroxyl radicals, nitric oxide, carbon-centered free radicals, singlet oxygen lipid-free radicals, and peroxynitrite [84,86]. Free radicals are naturally produced in the body by several metabolic reactions. A complex, enzymatic antioxidant defense system exists in order to minimize their potential damage, and is coupled with a non-enzymatic antioxidant system, which includes reduced glutathione (GSH), ascorbic acid, α-tocopherol, β-carotene, and polyphenols [87].

Several studies indicate phenolic compounds as the main components responsible for the protective effects of plant-based diets against aging-associated cognitive decline and neurodegenerative disorders such as AD, and insist on a simultaneous improvement of cognitive performance [3,4,65,88]. Polyphenols’ acute cognitive effects are, on the contrary, attributed to increased cerebral blood flow (CBF) [47]. The “direct” antioxidant activity of flavonoids derives from their chemical structure, in particular, the phenolic hydroxyl groups present on the B ring, and when present, a conjugated aromatic system [89].

The molecular mechanisms through which polyphenols exert neuroprotection can be complex and can involve properties of these molecules other than antioxidation, such as their antiaggregation effects on amyloids [89], or different mechanisms, e.g., modulation of specific cellular signaling pathways. Many of these complex pathways are involved in cognitive processes and are summarized in Table 4.

Even if the main dietary sources of polyphenols are fruits (especially berries), legumes, tea, coffee, wine, beer, cocoa, grains, nuts, oilseeds, herbs and spices [84,90], the dietary use of not all of these foods has been described in relation to human cognition.

Among all nuts, walnuts contain the largest amount of free and total polyphenols, followed by Brazil nuts [4]. Unfortunately, studies on the relation between nut consumption and cognitive health are limited, but nut intake has been shown to have an overall positive impact on cognitive functions. Available studies with nuts have shown improved antioxidant status, global cognition and perception, verbal fluency, and delayed onset of cognitive impairment in older adults and improved inferential reasoning and cognitive functions in young adults [17,32,33,34,35,36,37,40].

Grapes contain a variety of phytochemicals, including phenolic acids and resveratrol [30,47,99]. Concord grapes (*Vitis lambrusca*) possess a peculiar richness and composition in polyphenols. Polyphenol-rich grape consumption exerts acute (mainly detected in youngsters) and chronic positive effects (especially in older adults) in human subjects [30,41,47]. For example, resveratrol is known to promote antiaging effects and protect against beta-amyloid peptide [41]. It can increase the expression of nuclear factor (erythroid-derived 2)-like 2 (Nrf2) related genes [43] and Sirtuin 1 (Sirt1), which has neuroprotective effects [59]. Potential mechanisms underlying the effects of grapes on cognitive health could also include the modulation of CBF, the modulation of glucose metabolism and the inhibition of monoamine oxidase (MAO) activity [47].

Berries contain large amounts of anthocyanins, flavonols—like quercetin, myricetin and kaempferol—proanthocyanidins, ellagitannins, and phenolic acids [4,46]. *Ribes nigrum* (black currant) is a rich source of anthocyanins and flavonols. Studies investigating different berry types provided data demonstrating an improvement, significant in most of them, in cognitive function, in particular in memory [46,47,48,49,57,71]. Their beneficial effects have been attributed not only to their antioxidant action, but also to a decrease in blood pressure, mitigation of neuroinflammation, protection against cardiovascular risk, interaction with gut microbiota, increased neurogenesis (particularly in the hippocampus), and modulated glucoregulation [4,47,48].

Cherries contain large amounts of anthocyanins and are a good source of flavan-3-ols and flavonols [69]. Tart cherries have higher amounts of antioxidants, so they are placed at rank 14 out of the top 50 highest antioxidant-containing foods [51]. Unfortunately, the number of available studies of the effects of cherries on human cognitive health is limited, but chronic consumption of cherry juices has been shown to improve memory, learning, visual sustained attention, and verbal fluency in older adults [52,69]. Cherries have been shown to scavenge free radicals, to have anti-inflammatory effects, and to improve antioxidant defenses in older adults [50,51,52,69]. Tart cherry juice improved brain perfusion in healthy patients, which could be possibly attributed to increased NO synthase [52].

Pomegranates are rich in anthocyanins and hydrolysable tannins, especially glycosides of ellagic acid [4], but the currently available data are insufficient to postulate a precise mechanism of action for pomegranate juice on memory. Yuan and colleagues [100] found that although polyphenols are unable to cross the blood–brain barrier (BBB), urolithins, produced by gut bacteria from pomegranate polyphenols, are able to cross, for which pomegranate metabolites could have an impact on memory neural circuits. Evidence from intervention studies on memory enhancement in human, although limited, suggests a role for pomegranate juice in augmenting memory function through task-related increases in functional brain activity [53,54].

Oranges are a rich source of flavanones (hesperidin), able to cross the BBB, and flavonols (rutin and quercetin). Rutin has been shown to decrease and reverse amyloid β-protein fragment 25–35 (Aβ25–35) fibril formation and in vitro aggregation, prevent mitochondrial damage, reduce OxS marker generation, and enhance antioxidant enzymes. Its action is related to its aglycon, quercetin, a metabolite of rutin, absorbed mainly in the colon [55]. Furthermore, orange juice appears to benefit cognitive functions and psychomotor performance across age groups, the effects of which appear associated with an improved CBF [57]. Although the antioxidant activities of orange phytochemicals are likely to be crucial in the orange-associated neuroprotection, the mechanisms involved remain to be clearly elucidated [56,57].

Whole apples provide around 184 mg of total quercetin glycosides and 180 mg of epicatechins [76]. Unfortunately, studies on cognitive effects of apple consumption are scarce. It remains unclear whether longer supplementation with apple juice would help maintain cognitive performance [70,76].

Onion is an excellent source of flavonoids, above all quercetin. It upregulates SIRT-1, which has neuroprotective effects, and has anti-dysglycemic activities. It has been suggested that the ingestion of quercetin-rich onions improves cognitive function and reduces cognitive decline in elderly people [59].

Carnosic acid and its oxidized form, carnosol, are considered the main antioxidants of rosemary [72]. These two phenolic diterpenes have been shown to exert neuroprotective effects in vitro on human brain cells. Carnosic acid can suppress the production of amyloid-β 1–42, via the induction of metalloproteinases, in SH-SY5Y human neuroblastoma cells [61]. Data show a significant effect of rosemary on memory function, compared to placebo: beneficial for the lowest dose but detrimental at the highest dose tested, although the real-world impact of regular consumption of rosemary cannot be addressed [72].

Cocoa is one of the most flavonoid-rich foods, containing large amounts of flavan-3-ol, epicatechin, catechin, and additional oligomeric procyanidins [4]. Its flavonoids have been shown to benefit cognition across all ages and exert acute and chronic effects [47,57,62,78,101]. Cocoa flavonoids have been implicated in enhanced neurogenesis and neuroplasticity by stimulating brain-derived neurotrophic factor (BDNF) synthesis [4]. The associated benefits also include improved neurovascular function [47,62]. Flavanol-rich cocoa has been shown to increase CBF and the blood oxygenation level dependent (BOLD) response to task switching, and improve endothelial dysfunction by increasing the synthesis of NO by blood vessels, as seen in coffee intake, although these mechanisms need further clarification [101].

Coffee and tea are rich sources of phenolic compounds, such as hydroxycinnamic acids, 4-caffeoylquinic acid, and 5-caffeoylquinic acid; and some catechols [4]. The most abundant polyphenol in coffee is chlorogenic acid [63]. Paper-filtered coffee could reduce the generation of formamidopyrimidine DNA glycosylase (FPG), a marker of DNA oxidative damage. Interestingly, in other investigations, metal or unfiltered coffee was able to reduce a wider range of purine oxidative damage markers, including SOD and GST [102]. Although data on cognitive effects are still inconsistent [63,82], it has been hypothesized that coffee could exert positive effects on cognitive function not only by its antioxidant contents, but also because of its anti-diabetic activities, possibly due to its elevated magnesium content, which would increase insulin sensitivity. Insulin resistance is a well-known risk factor for accelerated cognitive decline and memory impairment, and diabetes is linked to a higher risk of dementia. Actually, AD has been also defined as brain-specific or type 3 diabetes, since one of the hallmarks of AD is also a reduction of the level of the cerebral insulin signaling, which is possibly related to hyperinsulinemia [3,103]. Another theory is that the neuroprotective effects of coffee could be partly mediated by ferulic acid—a strong antioxidant and phase 2 inducer—since its plasmatic levels rise after ingestion of coffee, due to gastrointestinal metabolism of chlorogenic acid [64].

Green tea is well known for its notable content of flavanols (catechins, epicatechins, and procyanidins), and, at a lesser extent, phenolic acids as well. A cup of green tea is estimated to contain an average of 67 mg of flavan-3-ols [66]. For green tea, data derived from observational and intervention studies are still inconsistent [63,80,81,82]. Furthermore, green tea has also been shown to benefit mood [57]. Epigallocatechin gallate (EGCG) activates SIRT1-dependent peroxisome proliferator-activated receptor-γ coactivator (PGC-1α), nuclear respiratory factor-1 (NRF-1), and mitochondrial DNA content [58]. It is also able to induce the expression of *Nrf*-2 [43] and to competitively inhibit acetylcholinesterase (AChE) and butyrylcholinesterase (BuChE), a feature that is especially attractive for AD patient treatment [57].

The above results support the hypothesis that chronic consumption of both coffee and tea (especially tea—particularly green tea) could lower the risk of cognitive impairment. Interestingly, evidence suggests this relation is linear for green tea, and biphasic and dose-dependent for coffee [4]. One possible explanation is that high coffee intake can cause the elevation of homocysteine, which is neurotoxic [104].

As a matter of fact, age may influence the effects of polyphenols on cognitive outcomes. Young and middle-aged adults appear to respond mostly with improving executive function, working memory, and psychomotor processing, while in older adults, effects on episodic memory, which is typically weaker in these subjects, appear more prevalent. Although CBF appears to be the common associated factor, the correlations between improved CBF and cognitive outcomes are inconstant and need further elucidation [4,44,57,76]. Besides polyphenolic compounds’ antioxidant effects, cognitive improvements can be related to other physiological changes, such as altered rates of glucose absorption and inhibition of MAO, which are also attributable to plant polyphenols and could play important roles in the observed cognitive response [57]. The majority of the observed cognitive improvements match peaks in plasma and cerebral metabolite levels [4,44,57,76]. Moreover, with a closer look at the impacts of micronutrients and single dietary compounds, there is also room for speculation as to the roles of some molecules absent in plant foods that might be detrimental to general and cognitive health, such as casein-derived opioids; neurotoxins such as PCB, dioxins, and others; and antibiotics—all contained in animal products [105].

## 5. Conclusions

The foods described in our review have been shown, although not consistently, to exert protective effects on or even improve cognitive functions, globally or for some specific domains. Acute effects are registered within hours of their consumption, while chronic benefits could appear after a few weeks or up to decades of regular intake. These benefits could be more relevant for older adults than younger subjects. The timing of the effects could depend on the sources and types of polyphenols; biochemically, their rates of absorption and bioavailability, rates of metabolism, and aptitude at crossing the BBB; or even the composition of the diet or the meal. It should nevertheless be kept in mind that the results of studies of this kind can be biased by many factors, including the heterogeneity of designs and non-dietary influences, necessitating caution when considering any kind of association found. Moreover, it is clear that food antioxidants exert their effects on cognition through antioxidative mechanisms and other mechanisms.

Since different dietetic patterns and specific foods appear to affect particular cognitive features, it is important to assess and differentiate multiple cognitive domains in future studies of diet and cognition. In order to plan future investigations with respect to phytocompounds and neuroprotection, it will be crucial to understand the underling mechanisms of neurodegeneration. More long-term, prospective studies in this field are warranted.

## Figures and Tables

**Table 1:** The major phytochemicals in dietary plant food (in order of food presentation).

Plant Food	Phytochemicals	Effects and Mechanisms	References
Nuts	Tocopherols, PUFA, especially α-linolenic acid (18:3 n–3, ALA, the plant-origin ω-3 fatty acid), carotenoids, tannins, naphthoquinones, phenolic acids (ellagic acid), phytosterols, polyphenols, melatonin, arginine, folates.	Antioxidant, anti-inflammatory. Improve antioxidant status, global cognition and perception, verbal fluency, delay onset of cognitive impairment in elderly subjects; improve inferential reasoning and cognitive functions in young adults.	[4,32,33,34,35,36,37,38,39,40]
Grapes	Stilbenes: trans-resveratrol, trans-resveratroloside, trans- pterostilbene, trans-picetannol, resveratrol. Phenolic-acids: caffeic acid, ferulic acid, p-coumaric acid, vanillic acid, gallic acid. Flavonoids: quercetin, kaempherol etc.	Antioxidant, anti-inflammatory. Protect against beta-amyloid peptide formation, increase the expression of Nrf2-related genes, modulate cerebral blood flow (CBF). Modulate glucose metabolism and inhibit MAO and GABA-ergic activities. Protect cerebral metabolism. Acute and chronic effect on cognition.	[30,41,42,43,44]
Berries	Quercetin, myricetin, ellagic acid, stilbenoids, kaempferol, vitamin C, proanthocyanidins, ellagitannins and phenolic acids.	Antioxidant, anti-inflammatory (multiple pathways), increase enzymatic antioxidant defenses (GSH). Antimicrobial, improve glucoregulation, interaction with microbiota, CBF. Increase digit vigilance reaction time, executive and memory functions. Increase neurogenesis.	[4,45,46,47,48,49]
Cherries	Proanthocyanins, anthocyanins (cyanidin-3-glucosylrutinoside, cyanidin-3-rutinoside, cyanindin-3-glucoside, and their agylcone, cyanidin), flavonols, melatonin.	Antioxidant, anti-inflammatory. Reduce the I/R-induced F2-isoprostane response and basal urinary excretion of oxidized nucleic acids (8-hydroxy-29-deoxyguanosine, 8-hydroxyguanosine or 8-OHdG).	[50,51,52]
Pomegranate	Ellagitannin, anthocyanins, flavan 3-ols, flavonols, catechins.	Antioxidant, anti-inflammatory. Pomegranate juice inhibited inflammation and amyloidogenesis in IL-1β-stimulated SK-N-SH cells.	[53,54]
Oranges	Flavanones (hesperidin), flavonols (rutin and quercetin).	Anti-inflammatory, anticancer, antithrombotic, cytoprotective and vasoprotective, inhibit Aβ25–35 fibril formation and aggregation in vitro, prevent mitochondrial damage, reduce OxS marker and proinflammatory cytokine (IL-1 β, TNF-α) generation, enhance antioxidant enzymes, improve CBF, and cognitive function.	[55,56,57]
Onion	Flavonoids (quercetin), phytosterols, saponins and sulphur-containing compounds, like N-acetylcysteine (NAC), S-methyl-L-cysteine, and S-propyl-L-cysteine sulfoxide.	Antioxidant, anti-inflammatory, anti-viral. Up-regulation of SIRT-1 and gluco-regulation (quercetin). Helps protect against cognitive decline.	[58,59]
Rosemary	Carnosic acid and carnosol (diterpens) are the two major antioxidants.	Antioxidant, anti-inflammatory. The two diterpens can induce Nrf2 and phase II detoxifying enzymes. Neuroprotective effects in vitro on human brain cells. AChE and BChE inhibition, DA neuron protection; metalloproteinase induction (carnosic acid).	[43,59,60,61]
Chocolate	Large amounts of the flavan-3-ol epicatechin, catechin, oligomeric procyanidins.	Antioxidant, anti-inflammatory. Stimulation of BDNF synthesis (neurogenesis, neuroplasticity), improvement of neurovascular function, CBF, BOLD.	[4,47,62]
Coffee	Rich source of caffein, and phenolic acids, especially chlorogenic acids, ferulic acid, hydroxycinnamic acids, 4-caffeoylquinic acid and 5-caffeoylquinic acid.	Antioxidant, anti-inflammatory, phase II detoxifying enzymes. Improves IR (antidiabetic). Acute and chronic effects on cognition, with J shaped curve on dementia for chronic consumption.	[4,63,64]
Tea	Major source of catechins (epicatechins and procyanidins) especially EGCG, theaflavin, caffeine.	Antioxidant, anti-inflammatory. EGCG inhibits AChE and BuChE, modulates the accumulation of amyloid fibrils and α-synuclein in vitro. Modulation of pro-apoptotic genes. Calming effect. Lipoxygenase inhibitor.	[57,58,65,66,67]

**Table 2:** Studies included in the final pool for review.

Authors, Year	Food	Study Design	Country	Subject Numbers and Characteristics	Sex	Age (Years)	Results	Efficacy
Arab & Ang, 2015 [37]	Nuts	Cross-sectional study of 2 groups of subjects	USA	12,693, US civilian population (excluded who had a stroke or a neurological disorder), aged 20–59 years (5356) and >60 (7337).	7070 F	37.4, 70.1 mean	Significant, positive associations between walnut consumption and cognitive functions among all adults, regardless of age, gender or ethnicity.	SE
Bondonno et al., 2014 [76]	Flavonoid-rich apples and spinach	Crossover RCT	Australia	30 healthy volunteers; 4 intervents in random order: (1) control: low flavonoid apple control (C) and low nitrate C; (2) apple: high flavonoid apple active (A) and low nitrate C; (3) spinach: low flavonoid apple C and nitrate-rich spinach A; (4) apple + spinach: high flavonoid apple A and nitrate-rich spinach A.	24 F	47.3 mean	No significant effect was observed on cognitive function.	NE
Bookheimer et al., 2013 [54]	Pomegranate juice	Placebo RCT	USA	28 non-demented, elderly subjects; 15 pomegranate juice, 13 placebo flavor-matched drink.	21 F	62.5 mean	Pomegranate group showed a significant improvement in memory scores (recall measure and long-term retrieval) and increased task-related brain activation in healthy elderly subjects.	SE
Bowtell et al., 2017 [49]	Blueberry concentrate juice	Double-blind placebo RCT	UK	26 healthy elderly subjects, 12 blueberry concentrated juice, 14 isoenergetic placebo.	13 F	68.2 mean	Non significant improvement in working memory (2-back test) in the blueberry versus the placebo group.	NSE in cognitive subsets
Cardoso et al., 2016 [35]	Brazil nuts	Placebo RCT	Brazil	31 subjects >60 years with MCI: 16 brazil nuts, 15 control group.	22 F	77.7 mean	Changes in the total score not significantly different between groups. Statistically significant improvement only in verbal fluency and constructional praxis compared with control group.	NSE total score/SE in cognitive subsets
Chai et al., 2019 [52]	Montmorency tart cherry juice	Placebo RCT	USA	37 adults aged 65–80 years with normal cognitive function enrolled, 34 completed: 17 tart cherry juice, 17 control juice.	20 F	65–80	Significantly higher memory scores and learning task performances in the tart cherry compared to the control group. Significant improvement of sustained attention and spatial working memory in the within-group in tart cherry juice compared with corresponding baseline values.	SE
Crichton et al., 2016 [78]	Chocolate	Cross-sectional study	Australia	968 non demented community dwelling; frequency intake of chocolate assessment (never/rarely or at least once/wk).	558 F	23–98, 61.9 mean	More frequent chocolate consumption was significantly associated with better performance in all tests, with the exception of working memory.	SE all domains except working memory
Dai et al., 2006 [74]	Fruit and vegetable juice	Prospective study (8 years: 4 follow-up waves, each 2 years apart)	USA	1589 non demented aged >65 years.	864 F	>65, 71.8 mean	The risk for probable AD was significantly reduced among people who drank fruit and vegetable juices 3 or more times per wk, compared with those who drank these juices less than once per wk.	SE
Eskelinen et al., 2009 [63]	Green tea and coffee	Prospective study (follow-up: 21 years)	Finland	1409 elderly subjects randomly selected from the survivors of a population-based cohort previously surveyed at midlife visit.	874 F	71.3 mean	Moderate coffee consumption at midlife may decrease the risk of dementia/AD later in life. No association between tea consumption and risk of dementia (small sample)	SE for coffee/ NSE for tea
Godos et al., 2020 [75]	Flavonoid	Prospective study (follow-up: 24 mo)	Italy	883 subjects divided by quartiles of total polyphenol intake: Q1 (*n* = 184), Q2 (*n* = 237), Q3 (*n* = 253) and Q4 (*n* = 209).	382 F	64.9 mean	Significant inverse association between higher dietary intake of total flavonoids and impaired cognitive status. Among individual subclasses of flavonoids, flavan-3-ols, catechins, anthocyanins and flavonols, and among individual polyphenols only quercetin, were associated with cognitive health.	SE
Haller et al., 2018 [79]	Coffee, wine and chocolate	Prospective study (follow-up: 3 years)	Switzerland	145 community-based elderly individuals with preserved cognition aged 69–86 years.	81 F	69–86, 73.8 mean	Moderate consumption of caffeinate (coffee and chocolate) was related significantly to better cognitive outcome. In contrast, increased consumption of wine was related to an unfavorable cognitive evolution.	SE caffeinate; NE wine
Haskell-Ramsay et al., 2017 [47]	Purple grape juice	Double-blind, counterbalanced-crossover placebo RCT	UK	20 healthy young adults.	13 F	18–35	Purple grape juice significantly improved reaction time on a composite attention measure compared to placebo.	SE
Ide et al., 2016 [80]	Green tea	Placebo RCT	Japan	27 elderly nursing home residents with cognitive dysfunction, 17 assigned to green tea and 16 to placebo.	29 F	84.8 mean	Changes in cognitive scores after 1 year of green tea consumption were not significantly different compared with that of the placebo group.	NE
Kean et al., 2015 [56]	Orange juice	Double-blind, crossover, placebo RCT	UK	37 healthy elderly subjects aged 60–81: high flavanone 100% juice and low flavanone control juice.	24 F	60–81, 66.7 mean	Global cognitive function was significantly better after 8 wk of consumption of high flavanone (HF) orange juice relative to 8 wk of consumption of the control low-flavanone (LF) juice; better performance in sustained attention and episodic memory when the HF drink rather LF drink was consumed during the first arm.	SE
Kent et al., 2017 [69]	Cherry juice	Placebo RCT	Australia	49 elderly subjects with mild-to-moderate AD recruited, 42 completed: 21 cherry juice, 21 (controls) apple juice.	not specified	79.7 mean	Significant improvements in verbal fluency, short-term memory and long-term memory in the cherry juice group. No significant improvements from baseline in the control group.	SE
Krikorian et al., 2010 [30]	Concord grape juice	Double-blind, placebo RCT	USA	12 elderly subjects with acquired early memory decline (not dementia); 5 Concord grape juice 100%, 7 placebo juice.	4 F	78.2 mean	Significant improvement in measures of verbal learning and non-significant enhancement of verbal and spatial recall.	SE in cognitive subsets
Krikorian et al., 2010 [48]	Wild blueberry juice	Double-blind, placebo RCT	USA	9 elderly subjects with early memory changes in wild blueberry juice; 7 control group and beverage as in Krikorian study [30].	4 F	76.2 mean	Statistically significant improvement of memory function (paired associate learning and word list recall).	SE
Kuriyama et al., 2006 [82]	Green, black or oolong tea	Cross-sectional study	Japan	1003 elderly subjects >70 (excluding subjects with missing data on body weight, height, blood glucose concentrations, blood pressure, or depressive symptoms)	531 F	74 mean	Regular green tea consumers were less likely to develop cognitive impairment. On the other hand, black or oolong tea consumption was not correlated with significantly lower risk of developing a cognitive impairment	SE green tea/NSE other teas
Lee et al., 2017 [41]	Grape freeze-dried powder	Double-blind, placebo RCT	USA	10 elderly subjects with MCI randomized to consume an active grape formulation (*n* = not specified) or a matched placebo formulation (*n* = not specified).	5 F	66–82, 72.2 mean	Supplementation with grapes did not change neuropsychological battery measures but showed a protective effect on brain metabolism.	NE/NSE in subgroup
Miller et al., 2018 [71]	Tifblue blueberry lyophilized	Double-blind, placebo RCT	USA	37 elderly subjects with cognitive decline as the ages: 18 blueberry, 19 control beverage.	24 F	60–75	Subjects in the blueberry group showed, relatively to controls, significantly reduction in reaction times, fewer repetition errors in long term memory test and reduced switch cost on a task-switching test across study visits.	SE
Ng et al., 2008 [81]	Tea and coffee	Cross-sectional study plus prospective study (follow-up 1–2 years, median 16 mo)	China	Cross sectional on 2194 and prospective study on 1438 healthy adults >55 years.	1323 F	65.6 mean	More frequent tea consumption, of any kind, was associated with lower risk of cognitive impairment and cognitive decline. No association btw coffee and cognitive impairment	SE for teas; NE for coffee
Nilsson et al., 2017 [46]	Mixture of berries beverage	Crossover RCT	Sweden	40 healthy 50–70 years old volunteers, 20 berry beverage and 20 control 5 wks, 5 wks interval then reverse 5 wks.	30 F	50–70	Subjects performed significantly better in the working memory test after the berry beverage compared to after the control beverage. No significant effects on the other test variables were observed.	SE only 1 domain
Nishimura et al., 2017 [59]	Onion powder	Double-blind, placebo RCT	Japan	50 healthy or with MCI subjects, randomized: 25 quercetin-rich onion powder and 25 a placebo onion powder without detectable quercetin.	25 F	65–84	No differences in MMSE and cognitive impairment rating scale scores between the two groups. Only in younger subjects the MMSE scores were significantly higher in the active test food group than in the placebo food group at wk 24.	NE/SE in subgroup
Nooyens et al., 2011 [73]	Fruit and vegetable	Prospective study (follow-up: 5 years)	The Netherlands	A general population sample of 2613 subjects (excluding those who reported having experienced a stroke); habitual amount and frequency of fruit and vegetable intake studied in association with baseline and change in cognitive function.	1325 F	43–70	Total intakes of fruits, legumes and juices were not associated with baseline or change in cognitive function. High intakes of some subgroups of fruits and vegetables (such as nuts, cabbage and root vegetables) were associated with significantly better cognitive function at baseline and/or smaller decline in cognitive domains.	SE subgroups of fruits and vegetables
O’Brien et al., 2014 [36]	Nuts	Prospective study (follow-up: 6 years)	USA	15,467 women >70 years without a story of stroke.	15,467 F	74.3 mean	Higher total nut intake over the long term was associated with significantly better cognitive global performance at older ages.	SE
Pengelly et al., 2012 [72]	Rosemary	Double-blind, repeated-measures, crossover, placebo RCT	USA	28 healthy and non-smoking elderly subjects aged 65–90 years cognitively tested 1, 2.5, 4, and 6 h following a placebo and four different doses of dried rosemary.	20 F	65–90, 75 mean	Significant improvement in measures of “speed of memory” compared with placebo with the lowest dose (750 mg) of rosemary, worsening with highest dose (6000 mg).	SE with lowest dose
Pribis et al., 2012 [40]	Walnuts	Double-blind, crossover, placebo RCT	USA	47 college students: 23 walnut-placebo group, 24 placebo-walnut group.	not specified	18–25, 20.6 mean	No significant increases were detected for non-verbal reasoning or memory on the walnut supplemented. Significant, moderate effect size increase in inferential reasoning.	NE/SE in cognitive subsets
Remington et al., 2010 [70]	Apple juice	Open-label pilot study	USA	21 nursing home residents with moderate-to-late-stage AD, no control group.	not specified	72–93	No change in cognitive performance; significant improvement reported by caregivers in behavioral and psychological symptoms associated with dementia.	NE
Sala-Vila et al., 2020 [32]	Walnuts	Dual center, single blind, parallel-group RCT	USA/Spain	657 cognitively healthy elders, 336 walnut diet and 321 control diet.	439 F	63–79	Walnut supplementation for 2 years had no effect on cognition in healthy elders; analyses by site suggest that walnuts might delay cognitive decline in subgroups at higher risk.	NE/NSE in subgroup
Siddarth et al., 2020 [53]	Pomegranate juice	Double-blind, 2-group parallel RCT	USA	200 subjects with normal aging or MCI: 98 pomegranate juice, 102 placebo drink.	174 F	50–75	Daily consumption of pomegranate juice has shown to stabilize the ability to learn visual information over a 12-mo period.	NSE
Valls-Pedret et al., 2015 [33]	MD+ olive oil/nuts	Parallel-group RCT	Spain	334 subjects at high cardiovascular risk but without cardiovascular disease.	233 F	55–80	A Mediterranean diet (MD) supplemented with olive oil or nuts is associated with significant improvement in cognitive function.	SE

Abbreviations: MCI: mild cognitive impairment; MMSE: mini mental state examination; NE: no efficacy; NSE: not significant efficacy; RCT: randomized controlled trial; SE: significant efficacy.

**Table 3:** A simple classification of naturally occurring plant polyphenols.

	Classes		Phenolic Compounds	Sources
FLAVONOIDS	Anthocyanins		Aurantidin, cyanidin, luteolinidin, rosinidin, petunidin, malvidin, peonidin, etc.	Fruit, vegetables, nuts, medicinal plants, especially cranberries, black currants, red grapes, raspberries, strawberries, blueberries and blackberries.
	Isoflavanoids	Isoflavons	Genistenin, daizenin, glycitein.	Legumes, medicinal plants, especially soy, but also lupin, fava beans, kudzu.
		Isoflavans	Equol.	
	Flavanols, flavan-3-ols or catechins		Catechins, epicatechins, epigallocatechin (EGC), epicatechin gallate (ECG), epigallocatechin gallate (EGCG).	Fruit, medicinal plants and others, especially tea, chocolate.
	Flavanones		Eriodictyol, narigenin, hesperitin, naringenin, abyssinones, hesperidin.	Fruit, medicinal plants and others, especially citrus fruits (orange, lemon) and grapes.
	Flavonols		Kaempferol, myricetin, fisetin, rutin, quercetin.	Fruit, medicinal plants and others, especially onions, kale, lettuce, tomatoes, apples, grapes and berries.
	Flavones		Apigenin, tangeretin, baicalein, rhoifolin.	Fruit, vegetables, medicinal plants, especially celery, parsley, red peppers, chamomile, mint, ginkgo biloba, peels of citrus fruits.
	Chalcones			Fruit, vegetables, nuts, medicinal plants, especially tomatoes, pears, strawberries, bearberries and certain wheat products.
NON FLAVONOIDS	Phenolic acids	Hydroxybenzoic acids	Benzoic acid, gallic acid, salicylic acid, vanillic acid, ellagic acid.	Fruits, vegetables, medicinal plants, grains and others, especially coffee, tea, berries, pomegranate.
		Hydroxycinnamic acids	Caffeic acid, chlorogenic acid, ferulic acid, sinapic acid, cumaric acid, gallic acid, rosmarinic acid.	
	Curcuminoids		Curcumin.	Tumeric
	Stilbenes		Resveratrol.	Fruit, nuts, medicinal plants and others, especially grapes and red wine.

Adapted from Panche et al., 2016 [83]; Ebrahimi & Schluesener, 2012 [84], Kumar & Goel, 2019 [85], Nishimura et al., 2017 [59], Rajaram et al., 2019 [4], and Pistollato et al., 2018 [3].

**Table 4:** Polyphenols and neuroprotection: hypothesized pathways involved.

Hypothesized Effect	Overview
Direct oxidants scavenge	Scavenge superoxide and hydroxyl radicals, peroxyl radicals, NO, carbon-center free radicals, singlet oxygen and lipid free radicals, and peroxynitrite [84,86].
Modulation of cellular signaling pathways	Neuroprotection via the activation of pro-survival pathways (ERK1/2, PI3K/Akt, PKC), and inhibition of pro-apoptotic pathways (JNK, p. 38) [84,86,90].
Mitigation of mitochondrial dysfunction	Modulating mitochondrial dynamics, function, and biogenesis [19,58,90].
Induction of antioxidant and phase II detoxification enzyme expression	GSH, CAT, SOD, CYP 450, glutathione S-transferase, NAD(P)H-quinone oxidoreductase, and UDP-glucuronosyl transferase (via Nrf2) [64,84,86,90,91,92]. This effect could be mediated by induction of cell’s adaptative response caused by polyphenol’s pro-oxidant activities [10,11,19].
Modulation of synaptic plasticity (SP)	Modulation of synaptic morphology, neuroreceptors, kinase activity, release of BDNF, CREB [42,47,93].
Anxiolytic effect	Via modulation of cortisol and GABA receptors and suppression of the activity of MAO [47,93,94,95].
Increased CBF	Improving vasodilatation, angiogenesis and functional hyperemia/neurovascular coupling (NVC) [42,62].
Metal chelation	Decreasing metal accumulation in neurons [84,90,91].
Anti-inflammatory activities	Attenuating the expression of several pro-inflammatory pathways (OX-2, iNOS, NF-kB, IL-6, IL-1β, IL-1α, TNF-α, p38 etc.), inhibiting microglial and astrocytic overactivation [86,90].
Anti-protein formation and aggregation	Modulating the formation and/or aggregation of α-synuclein protein, amyloid-β plaques, neurofibrillary tangles [86,90], and TAU proteins [86].
Glucose homeostasis/hypoglycemic effect	Improving insulin-sensitivity, and peripheral glucose uptake; stimulating insulin secretion, delaying glucose absorption, protecting pancreatic β-cells, suppressing glucose release from the liver, inhibiting aldose reductase and AGEs generation [91,96,97].
Protection against amyloid toxicity	Decreasing the accumulation of amyloid fibrils and α-synuclein as shown in vitro [41,89,98].
Catecholamines increase (dopamine and ACh)	Stimulating the tyrosine hydroxylase expression/activity, inhibiting AChE and MAO expression/activity, activating dopamine receptors [66,86,90,94].
